# Influence of nanoparticulated chitosan on the biomodification of eroded dentin: clinical and photographic longitudinal analysis of restorations

**DOI:** 10.1007/s10856-020-06487-2

**Published:** 2021-01-20

**Authors:** José Caetano de Souza, Antônio Cláudio Tedesco, Luandra Aparecida Unten Takahashi, Fabiana Almeida Curylofo-Zotti, Aline Evangelista Souza-Gabriel, Silmara Aparecida Milori Corona

**Affiliations:** 1 Department of Restorative Dentistry, Ribeirão Preto School of Dentistry, University of São Paulo, Ribeirão Preto, São Paulo, 14040-904 Brazil; 2 Department of Chemistry, Center of Nanotechnology and Tissue Engineering, Photobiology and Photomedicine Research Group, Faculty of Philosophy, Sciences and Letters of Ribeirão Preto, University of São Paulo, Ribeirão Preto, São Paulo, 14040-901 Brazil

## Abstract

To evaluate the influence of the pre-treatment with 2.5% nanoparticulate chitosan (2.5% NanoChi) solution on eroded dentin before the restorative dental treatment. The sample consisted of 22 patients (age between 33 and 52 years) with shallow or medium erosion lesions located in two homologous teeth. The teeth were randomly assigned according to dentin treatment: with 2.5% NanoChi and without with chitosan (control). The NanoChi were applied immediately after acid etching. The teeth were restored with Single Bond Universal (3 M) and Charisma resin (Kulzer). Analyzes were done using modified USPHS (retention, secondary caries, marginal adaptation, and sensitivity) and photographic (color, marginal pigmentation, and anatomical form) criteria at 7 days (baseline) and 1 year. Population demographics, Kaplan–Meier estimates and log-rank test (Mantel–Cox) were calculated for 1 year (*α* = 0.05). No significant difference was found in the survival rates between groups (*p* > 0.05) at 7 days and 1 year after treatment. After 7 days, 100% of the restorations were scored as *Alpha* on all criteria. After 1 year, 91% of the NanoChi restorations were scored as *Alpha* and 9% as *Charlie* for the retention, marginal adaptation, and anatomical form criteria, while 86% of the control restorations (without NanoChi) received the *Alpha* score and 14% received the *Charlie*. Secondary caries, sensitivity, color, and marginal pigmentation criteria were scored as *Alpha* in 100% of the restorations. The biomodification of eroded dentin with 2.5% NanoChi did not influence the survival of the restorations after 1 year. The application of 2.5% NanoChi on eroded dentin did not increase failures of resin restorations after 1 year and it can be used as a pre-treatment solution.

## Introduction

Dental erosion is the irreversible and progressive loss of tooth structure caused by non-bacterial chemical processes with different etiological factors [1] that allow it to be classified as intrinsic and extrinsic. Intrinsic erosion occurs when teeth get into contact with gastric acid during recurrent vomiting or gastroesophageal reflux [2]. Extrinsic erosion is caused by exogenous acids from diet or drugs [3]. The prevalence of dental erosion has increased in industrialized countries [4], especially in overweight youth and children [5], due to the high intake of energy and isotonic drinks by athletes and citrus fruits [6].

The erosion process can affect both enamel and dentin. In enamel, the demineralization of the inorganic phase of the tooth exposes the prisms and reduces microhardness [7]. The dissolution of the peritubular portion and demineralization of intertubular dentin exposes the organic matrix, forming a rough and porous surface that increases the enzymatic degradation of collagen, therefore, impairing the adhesive interface in the restorative therapy [8, 9].

Dentin collagen degradation has generally been associated with poor clinical performance of composite resin restorations [10]. The adhesion between dental tissue and restoration is compromised in dentin exposed to mechanical or chemical challenges [11]. In eroded dentin, the high water content of collagen fibers hampers the adhesive infiltration and accelerates the degradation of the adhesive interface[12]. Previous studies suggest the use of natural solutions to modify the dental surface, such as chitosan, aiming to increase the mechanical resistance of collagen fibrils to degradation, providing support for the adhesive interface [13–15].

Chitosan, a hydrophilic biopolymer obtained by deacetylation of chitin [16, 17], is the most abundant polysaccharide in nature after cellulose [18], and has properties such as biocompatibility, bioadhesion, biodegradability, low human cell toxicity, and antimicrobial activity [17, 19], as well as chelating capacity [20], providing greater longevity in eroded dentin restorations [9]. Chitosan can increase the number of crosslinks between collagen fibers and neutralize metalloproteinases from dentin [16].

This study aimed to evaluate the influence of 2.5% w/w nanoparticulate chitosan (2.5% NanoChi) solution on eroded dentin biomodification at baseline (7 days) and final (1 year) periods before the restorative treatment, by clinical and photographic examination using modified United States Public Health Service (USPHS) criteria.

## Materials and methods

### Experimental design

For the experiment, 22 patients with erosive noncarious lesions located on the palatal or lingual surface of two homologous teeth (*n* = 22) were selected according to the randomized block design. The two teeth of each patient were divided into two groups: restoration with 2.5% NanoChi and without NanoChi (control). Restorations were evaluated clinically and photographically using modified USPHS criteria at baseline (7 days) and final (1 year) after treatment.

### Sample size calculation and ethical aspects

To determine the sample size, we used the power calculation function of the sample size calculation based on the protocol previously described in www.sealedenvelope.com, using the trial equivalent with the following parameters: *α* = 5%, power 90%, success percentage of control and experimental groups of 98% and equivalence limit of 15%, reaching a sample size of 19 restorations *per* group.

The project was approved by the local Research Ethics Committee (79949317.1.0000.5419) and was registered at the Brazilian Registry of Clinical Trials (RBR-22md2n). Each patient signed the informed consent form.

### Preparation of NanoChi solution

The preparation of 2.5% NanoChi was carried out at the Center for Nanotechnology and Tissue Engineering of the Ribeirão Preto School of Philosophy, Sciences and Letters (FFCLRP) from commercially available chitosan (Sigma-Aldrich, Saint Louis, MO, USA), low molecular weight (75–85% deacetylation).

Chitosan nanoparticles reached an average size of less than 300 nm. The polydispersity index varied between 0.311 and 0.422, which indicates nanoparticles of different sizes. The zeta potential is around +30 mV, considered strongly cationic. The presence of amino groups in the polymeric chain prevents the aggregation of the nanoparticles. Chitosan nanoparticles containing green tea reached an average size of less than 350 nm. The polydispersity index is smaller than 0.45, and the zeta potential is around +40 mV. Few variations along the 80-day period evaluated to indicate the long-term stability of the nanoformulation.

### Patients and teeth selection

Thirty-one patients aged between 33 and 52 years of both sexes were examined at the Dental Clinic of the Ribeirão Preto School of Dentistry. Patients underwent prophylaxis with pumice paste (SS White, Rio de Janeiro, RJ, Brazil), water and rubber cup (Jon, São Paulo, SP, Brazil) on smooth surfaces and Robinson brush (Jon, São Paulo, SP, Brazil) on occlusal surfaces, using a micromotor handpiece (Gnatus, Ribeirão Preto, SP, Brazil). Waxed dental floss (Aperibé, RJ, Brazil) was used on the proximal surfaces. Clinical examination was performed under adequate lighting after prophylaxis.

The inclusion criterion was the presence of at least two shallow or medium erosion lesions located on the palatal or lingual surface of homologous vital teeth. Tooth vitality was tested with the thermal test with Endofrost (Roeko, Langenau, Germany). Of the examined patients, 22 were selected. The medical history and dental charts were completed, and patients received individualized instructions on diet and oral hygiene. Patients with temporomandibular dysfunction, bruxism, teeth with pain, spontaneous tenderness, fistula, or edema were excluded from the study. The patients who were not selected for the study but needed restorative treatment were referred to our school’s dentistry clinic.

### Treatment of erosion lesions

Patients’ teeth were randomized into two groups using a computer spreadsheet and a random number generator available at the website http://randomnumbergenerator.intemodino.com/en. Prophylaxis was performed as previously described and initial photographs of the teeth were taken (buccal and palatal/lingual) (Canon EOS Rebel T2i 18.0 Megapixels, Cannon, Japan).

The color of the Charisma composite resin (Kulzer South America, Sao Paulo SP, Brazil) was then selected using the Vita 3D color scale (Wilcos do Brasil Industria e Comercio Ltda., Petrópolis, RJ, Brazil). Absolute isolation of the teeth was performed with a rubber dam (Madeitex, São Jose dos Campos, SP, Brazil) and clamps (Duflex, SS White, Rio de Janeiro, RJ, Brazil), according to the morphology of each tooth.

The enamel was acid-etched with 35% phosphoric acid for 30 s, and dentin was not etched. After the cavity was washed with water for 1 min, the excess water was removed with the suction cannula, and the surface dried with absorbent paper. In the experimental group, the 2.5% NanoChi was actively applied on the dentin with a single disposable microbrush (KGBrush, KG Sorensen, Cotia, SP, Brazil) for 1 min, followed by washing with water and drying with absorbent paper.

The layer of the Single Bond Universal adhesive system (3 M ESPE, Sao Paulo, SP, Brazil) was actively applied with a disposable microbrush (KGBrush, KG Sorensen, Cotia, SP, Brazil) for 10 s and light-cured (Gnatus, Ribeirão Preto, SP, Brazil). Charisma composite resins (Kulzer South America, São Paulo SP, Brazil) were used through the incremental restoration technique with a resin spatula, and each increment was light-cured for 20 s. Occlusal contact points were recorded with carbon paper (Angelus, Londrina, PR, Brazil) after tooth restoration. Premature contacts and occlusal interferences were removed using diamond burs (KG Sorensen, Cotia, SP, Brazil). Subsequently, diamond burs (KG Sorensen, Cotia, SP, Brazil) and polishing disks (TDV, Praxis, Santa Catarina, SC, Brazil) were used for finishing. The patients returned after 7 days for final polishing of the restoration with abrasive burs (Dentsply, Petrópolis, RJ, Brazil) and pumice paste (SS White, Rio de Janeiro, RJ, Brazil).

### Clinical and photographic analyzes of restorations

**Fig. 1:**
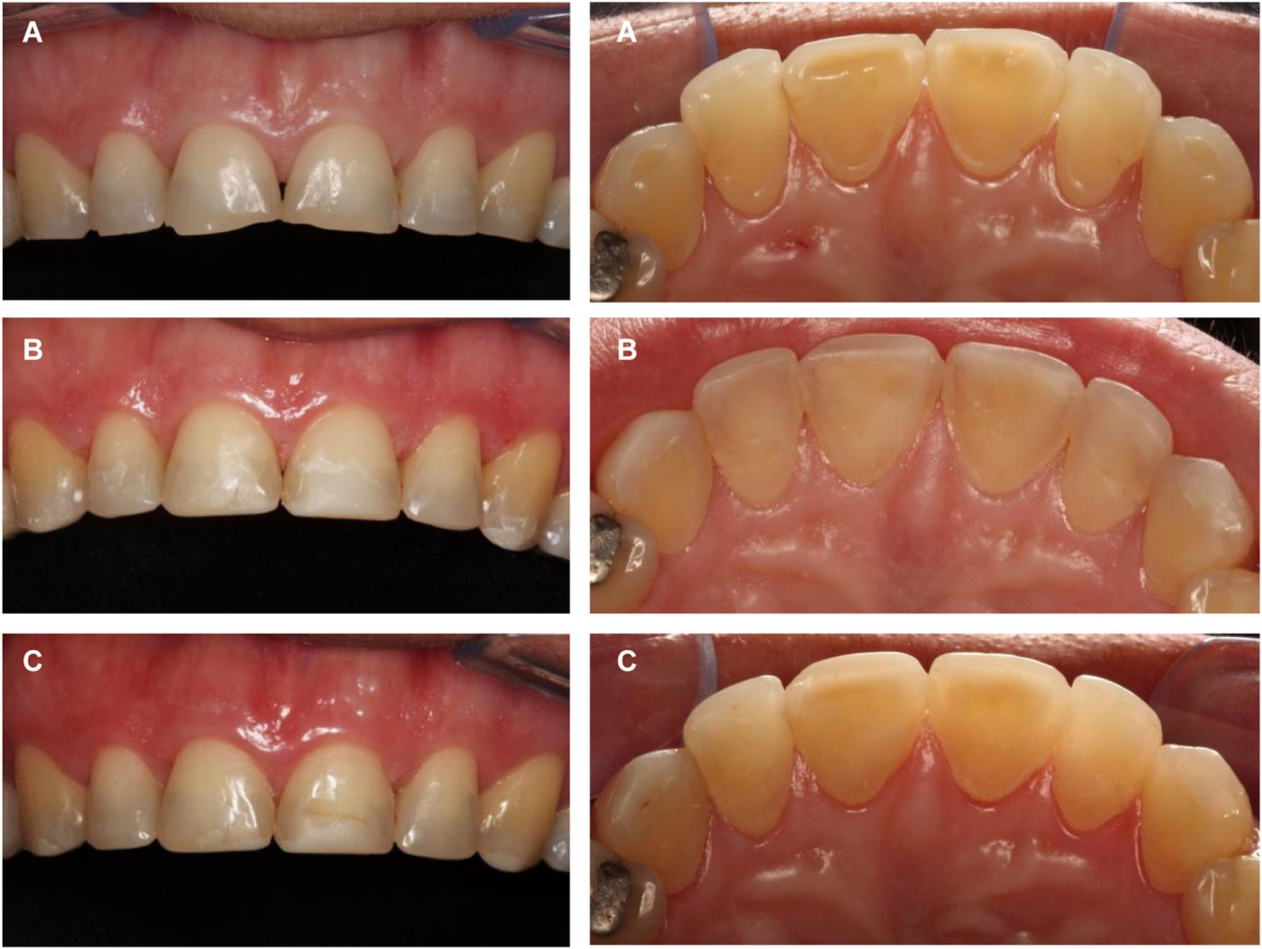
Clinical aspects of erosion lesions and restorations. **a** Buccal and palatal erosion lesions—clinical appearance (**b**) 7 days and (**c**) 1 year after treatment. The right central incisor was from the control group (without biomodification) and the left central incisor was from the test group (with 2.5% NanoChi)

Clinical and photographic analyzes of restorations were performed by three calibrated and experienced examiners long-term followed up evaluation at the 1-year interval, following the modified USPHS clinical (retention, secondary caries, marginal adaptation, and postoperative sensitivity) and photographic (restoration color, marginal pigmentation, and anatomical form) criteria [21]. The same dentist performed all restorations after training and calibration of the clinical protocol (Fig. 1). The clinical and photographic evaluations of the restorations (baseline and 1 year) were performed by three evaluators (dentists who did not participate in the restoration) after training and calibration.

The restorations were scored into *Alpha*—when the evaluated criterion had no problems, and the restoration was in perfect condition; *Bravo*—when the evaluated criterion had minor but clinically acceptable failures and *Charlie*—when the evaluated criterion had relevant failures and the restoration needed to be replaced (Table 1).

**Table 1:** Modified USPHS criteria used for the clinical and photographic analysis of restorations

Clinical criteria	Score	Photographic criteria	Score
Retention	A. No loss of restorative material B. Partial loss of restorative material C. Total loss of restorative material	Restoration color	A. Corresponds to adjacent dental structure in terms of color and translucency B. Slight change in color, shade, or translucency between restoration and adjacent tooth C. Clear color change and translucency
Secondary caries	A. No recurrence of caries B. With recurrence of superficial caries C. With recurrence of deep caries	Marginal pigmentation	A. No pigmentation along margin between restoration and adjacent tooth B. Slight pigmentation along the margin between the restoration and adjacent tooth C. Pigmentation present along restoration margin
Marginal adaptation	A. Perfectly adaptable with no visible margins B. Visible but clinically acceptable margin C. Marginal mismatch, clinical failure	Anatomic form	A. Restoration in continuity with existing anatomical form B. Restoration in discontinuity with anatomical form of tooth C. Loss of material by exposing dentin or restoration base
Postoperative Sensitivity	A. Missing stimulated sensitivity. B. Present and localized stimulated sensitivity. C. Present and diffuse stimulated sensitivity		

*A* Alpha, *B* Bravo, *C* Charlie scores

Intraoral photographs were taken at both time-points with the digital camera (Canon EOS Rebel T2i 18.0 Megapixels, Cannon, Japan) and a buccal photography mirror (Indusbello, Londrina, PR, Brazil), recording the buccal and palatal/lingual surfaces of the restored teeth, totaling two photos for each treatment. Photographic analyzes of restorations were performed by examiners viewing the images on a laptop screen, under the same environment and lighting.

### Data analysis

Statistical analysis was performed using SPSS statistical software for Windows version 25.0 (SPSS Inc., Chicago, IL, USA). Survival analysis by the Kaplan–Meier method and log-rank test (Mantel–Cox) were performed with the scores obtained during the clinical and photographic analyzes at both time-points. Cohen’s kappa coefficient was used to check the agreement level between operators.

## Results

Forty-four restorations (100% of the studied groups) were analyzed in 22 patients (7 men and 15 women) with a mean age of 40.5 years (range 33–52 years). The follow-up period was 1 year. Eight upper central incisors, ten upper lateral incisors, eight upper canines, six lower central incisors, six lower lateral incisors, and six lower canines were included in the study (Table 2). There was no statistically significant difference in the survival of restorations among men and women (*p* = 0.072), different age groups (*p* = 0.780), or position in the dental arch (*p* = 0.193).

**Table 2:** Profiles of the patients attended

Patient	Sex (M/F)	Age	Restored tooth (+)	Bone base	Lesion depth	Restored tooth (−)	Bone base	Lesion depth
1	M	33	21	Maxilla	Medium	11	Maxilla	Medium
2	F	47	21	Maxilla	Medium	11	Maxilla	Medium
3	F	39	21	Maxilla	Medium	11	Maxilla	Medium
4	F	52	21	Maxilla	Medium	11	Maxilla	Medium
5	F	40	22	Maxilla	Medium	12	Maxilla	Medium
6	M	34	22	Maxilla	Medium	12	Maxilla	Medium
7	F	51	22	Maxilla	Medium	12	Maxilla	Medium
8	M	37	22	Maxilla	Shallow	12	Maxilla	Shallow
9	F	46	22	Maxilla	Shallow	12	Maxilla	Shallow
10	F	39	23	Maxilla	Medium	13	Maxilla	Medium
11	M	38	23	Maxilla	Shallow	13	Maxilla	Shallow
12	F	52	23	Maxilla	Shallow	13	Maxilla	Shallow
13	F	47	23	Maxilla	Shallow	13	Maxilla	Shallow
14	F	40	31	Mandible	Medium	31	Mandible	Medium
15	F	45	31	Mandible	Shallow	31	Mandible	Shallow
16	M	38	41	Mandible	Shallow	41	Mandible	Shallow
17	M	40	32	Mandible	Medium	42	Mandible	Medium
18	F	46	32	Mandible	Shallow	42	Mandible	Shallow
19	F	39	32	Mandible	Shallow	42	Mandible	Shallow
20	M	41	33	Mandible	Medium	43	Mandible	Medium
21	F	42	33	Mandible	Shallow	43	Mandible	Shallow
22	F	47	33	Mandible	Shallow	43	Mandible	Shallow

With biomodification and (+) without biomodification (−) with NanoChi

*M* male, *F* female

No statistically significant difference (*p* > 0.05) was found between restorations with and without eroded dentin biomodification at baseline (7 days) and final (1 year) periods after treatment (*p* = 0.836). The evaluation of dental restorations obtaine an intra and inter examiner kappa of 1.0 (intra examiner kappa *A* = 1, *B* = 1 and *C* = 1; inter examiner kappa *A* × *B* = 1, *A* × *C* = 0.95, and *C* × *B* = 0.92), with perfect agreement among examiners.

After 7 days of the procedure, 100% of the restorations were scored as *Alpha* in all clinical (retention, secondary caries, marginal adaptation, and postoperative sensitivity) and photographic (restoration color, marginal pigmentation, and anatomical form) criteria.

After 1 year of the procedure, 91% of the restorations in the experimental group were scored as *Alpha* and 9% as Charlie for retention and marginal adaptation clinical criteria and anatomical form photographic criteria. In the control group, 86% of the restorations were scored as *Alpha* and 14% as *Charlie* for retention and marginal adaptation clinical criteria and anatomical form photographic criteria. Besides, 100% of the restorations were scored as *Alpha* for others clinical (secondary caries and postoperative sensitivity) and photographic (restoration color and marginal pigmentation) criteria in the experimental and control groups (Tables 3 and 4).

**Table 3:** Clinical analyzes at baseline (7 days) and final (1 year) periods after the restorative procedure

Treatment	Evaluation period		Retention			Secondary caries			Marginal adaptation			Postoperative sensibility		
With 2.5% NanoChi	Baseline (7 days)	*n* = 22 (%)	A	B	C	A	B	C	A	B	C	A	B	C
			22	–	–	22	–	–	22	–	–	22	–	–
			100	–	–	100	–	–	100	–	–	100	–	–
	Final (1 year)	*n* = 22 (%)	A	B	C	A	B	C	A	B	C	A	B	C
			20	–	2	22	–	–	20	–	2	22	–	–
			91	–	9	100	–	–	91	–	9	100	–	–
Without 2.5% NanoChi	Baseline (7 days)	*n* = 22 (%)	A	B	C	A	B	C	A	B	C	A	B	C
			22	–	–	22	–	–	22	–	–	22	–	–
			100	–	–	100	–	–	100	–	–	100	–	–
	Final (1 year)	*n* = 22 (%)	A	B	C	A	B	C	A	B	C	A	B	C
			19	–	3	22	–	–	19	–	3	22	–	–
			86	–	14	100	–	–	86	–	14	100	–	–

**Table 4:** Photographic analyzes at baseline (7 days) and final (1 year) periods after the restorative procedure

Treatment	Evaluation period		Restoration color			Marginal pigmentation			Anatomical form		
With 2.5% NanoChi	Baseline (7 days)	*n* = 22 (%)	A	B	C	A	B	C	A	B	C
			22	–	–	22	–	–	22	–	–
			100	–	–	100	–	–	100	–	–
	Final (1 year)	*n* = 22 (%)	A	B	C	A	B	C	A	B	C
			22	–	–	22	–	–	20	–	2
			100	–	–	100	–	–	91	–	9
Without 2.5% NanoChi	Baseline (7 days)	*n* = 22 (%)	A	B	C	A	B	C	A	B	C
			22	–	–	22	–	–	22	–	–
			100	–	–	100	–	–	100	–	–
	Final (1 year)	*n* = 22 (%)	A	B	C	A	B	C	A	B	C
			22	–	–	22	–	–	19	–	3
			100	–	–	100	–	–	86	–	14

Population demographics were computed and Kaplan–Meier survival estimates were calculated for 1 year. The survival of restorations at 1 year is 93% for teeth without biomodification and 95% for teeth with biomodification. These findings show that survival rates of restorations are high at 1 year after treatment.

At the 1-year interval, no significant difference in survival was noted between groups (*p* > 0.05) with and without eroded dentin biomodification. The success rate with 2.5% NanoChi was 95.5%, while in the control group (without NanoChi) the success rate was 93.2%.

## Discussion

The use of chitosan products in dentin tissues increases the surface resistance against collagenase degradation [14, 15] and contributes to forming a calcium phosphate layer on demineralized dentin [22]. Chitosan has great anti-erosive potential in enamel and dentin when associated with metal ions and fluoride [23]. The demineralized dentin models have shown that chitosan and calcium phosphate-based nanocomplexes favored the dentin remineralization [24]. At the same time, chitosan-based dentifrices significantly reduced dentin erosion by 24–67% (extrinsic conditions—citric acid, pH: 2.5) and 21–40% (intrinsic conditions—HCl/pepsin solution, pH: 1.6) [24, 25].

In enamel, calcium phosphate chitosan nanoparticles positively affect the remineralization process in a similar way to that in the oral cavity, but different from the remineralization that occurs by the fluoride action [26]. In dentin, phosphorylated chitosan increases the deposition of calcium and phosphate ions [22]. However, the chitosan solutions have limitations in reducing dentin surface loss after an erosion challenge compared to fluoride and metallic solutions [27]. The chitosan does not influence the microhardness and Ca/P percentage in dentin affected by residual caries [28].

In the present study, the application of the 2.5% NanoChi did not influence the quality and longevity of the restorations after 1-year based on the USPHS criteria, but the treatment with 2.5% NanoChi provided a higher percentage of flawless restorations. This result can be justified by only shallow and medium erosion lesions and one tooth surface (palatal or lingual) of patients who were not diagnosed with parafunctional habits or other symptoms resulting from dental erosion. Failures in adhesive restorations may be associated with untreated parafunctional habits that affect occlusions [29], such as bruxism and oral dysfunctions, which may affect oral vertical dimension and limit restorative treatment [30].

The use of the self-etching single bond universal adhesive system may have contributed to the good results obtained of the restorations of erosive lesions since this system chemically bonds to the dentin substrate [31, 32] and is stable in a wet environment [33]. A very important component in this adhesive is the 10-methacryloyloxydecylhydrogen phosphate (10-MDP). After self-etch, calcium ions of the hybrid layer upon contact with 10-MDP, chemically interact in nano-layers to form a salt (10-MDP-Ca), which increases the chemical adhesion of the adhesive system to the dental substrate [34] and favors clinical [35, 36] and laboratory [37, 38] results of restorations. As another factor that may have positively affected the results was the enamel-restricted acid etching done before applying the self-etching adhesive system, which increases the bond strength of enamel [39] and dentin [40].

In longer evaluation periods, more obvious failures are observed. Studies have shown that restorative treatments for noncarious cervical lesions, including erosion, present clinical failure after 2-year on average, with a significant deterioration in marginal adaptation and discoloration of the cavosurface margin [41]. Therefore, the fact that biomodification of eroded dentin with 2.5% NanoChi does not influence the clinical behavior of the restorations is favorable because adhesion depends on the adhesive system flow over the surface [31]. Thus, other positive aspects of chitosan as antimicrobial and chelating properties may be present without impairing adherence [42].

Furthermore, recent studies have shown the benefits of using chitosan. For example, chitosan–calcium aluminate support (CH–AlCa) combined with a dosage of 1α, 25-dihydroxyvitamin D3, provides a bioactive pulp cell microenvironment, which could be a potential tissue engineering system for direct pulp protection [43]. In another study, triclosan-associated chitosan adhesive resin showed greater antibacterial activity immediately and after 6-month, stabilizing the dentin-adhesive interface and maximizing long-term marginal sealing [44]. The chitosan–hydroxyapatite (C–HA) dentin conditioning enhances dentin surface wettability to facilitate tricalcium silicate sealant penetration and dentin tensile strength [45].

Due to the strong indications of the benefits of applying chitosan-based products on dentin, further longitudinal studies should be performed, assessing the long-term impact of this substance on the restorative treatment of eroded teeth.

## Conclusions

The biomodification of eroded dentin with 2.5% NanoChi did not directly influence the resin restorations based on clinical and photographic criteria after a 1-year follow-up. However, the application of 2.5% NanoChi on eroded dentin provided a higher percentage of flawless restorations. Even if the difference compared to the untreated group was not significant, further long-term studies are needed to support this outcome.
